# Methods for evaluating the impact of vertical programs on health systems: protocol for a study on the impact of the global polio eradication initiative on strengthening routine immunization and primary health care

**DOI:** 10.1186/1471-2458-12-728

**Published:** 2012-09-01

**Authors:** Svea Closser, Anat Rosenthal, Thomas Parris, Kenneth Maes, Judith Justice, Kelly Cox, Matthew A Luck, R Matthew Landis, John Grove, Pauley Tedoff, Linda Venczel, Peter Nsubuga, Jennifer Kuzara, Vanessa Neergheen

**Affiliations:** 1 Department of Sociology and Anthropology, Middlebury College, 306 Munroe Hall, Middlebury, VT, 05753, USA; 2 Department of Sociology and Anthropology, The Hebrew University of Jerusalem, Mount Scopus, Jerusalem, 91905, Israel; 3 ISciences, LLC, 61 Main St, Burlington, VT, 05401, USA; 4 Department of Anthropology, Oregon State University, 238 Waldo Hall, Corvallis, OR, 97331, USA; 5 Department of Anthropology, History, and Social Medicine, University of California, San Francisco, 3333 California Street Suite 340, Box 0646, San Francisco, CA, 94118, USA; 6 201 Munroe Hall, Middlebury College, Middlebury, VT, 05753, USA; 7 Infectious Diseases, Global Health Program, Bill & Melinda Gates Foundation, 500 Fifth Avenue North, Seattle, WA, 98102, USA; 8 Polio Program, Global Development Program, Bill & Melinda Gates Foundation, 500 Fifth Avenue North, Seattle, WA, 98102, USA; 9 Global Public Health Solutions, 4025 Runnymede Dr, Lilburn, GA, 30047, USA; 10 Department of Anthropology, Emory University, 1557 Dickey Dr, Atlanta, GA, 30322, USA

**Keywords:** Polio, Eradication, Vertical program, Routine immunization, Primary health care, Comparative ethnography, Health systems

## Abstract

**Background:**

The impact of vertical programs on health systems is a much-debated topic, and more evidence on this complex relationship is needed. This article describes a research protocol developed to assess the relationship between the Global Polio Eradication Initiative, routine immunization, and primary health care in multiple settings.

**Methods/Design:**

This protocol was designed as a combination of quantitative and qualitative research methods, making use of comparative ethnographies. The study evaluates the impact of the Global Polio Eradication Initiative on routine immunization and primary health care by: (a) combining quantitative and qualitative work into one coherent study design; (b) using purposively selected qualitative case studies to systematically evaluate the impact of key contextual variables; and (c) making extensive use of the method of participant observation to create comparative ethnographies of the impact of a single vertical program administered in varied contexts.

**Discussion:**

The study design has four major benefits: (1) the careful selection of a range of qualitative case studies allowed for systematic comparison; (2) the use of participant observation yielded important insights on how policy is put into practice; (3) results from our quantitative analysis could be explained by results from qualitative work; and (4) this research protocol can inform the creation of actionable recommendations. Here, recommendations for how to overcome potential challenges in carrying out such research are presented. This study illustrates the utility of mixed-methods research designs in which qualitative data are not just used to embellish quantitative results, but are an integral component of the analysis.

## Background

The impact of vertical health programs on the function of other healthcare activities has long been a topic of conversation and debate [1,2]. The need for an evidence base for how best to strengthen health systems amidst global resource constraints is increasingly clear [3-6], and solid evidence on the impact of vertical programs on health systems is needed. As is the case with systems research in other fields, interest in documenting change in health systems calls for new methods that go beyond previous analytical paradigms. Measuring the impact of global vertical programs requires gathering evidence on complex processes occurring in greatly diverse country and program implementation contexts. In this article, we describe a research protocol combining quantitative and qualitative research methods, and integrating a novel use of comparative ethnographies. This protocol was developed to assess the historical impact of the Global Polio Eradication Initiative (GPEI) on routine immunization (RI) and primary health care (PHC), and could be adapted and built upon to evaluate the strategies or impact of other vertical programs. Here, we describe the protocol, the challenges and opportunities it presented, and our recommendations to other researchers embarking on similar studies in the future.

Qualitative studies on the effects of vertical programs on health systems, including immunization programs, commonly include interview and document review components [7-10]. Quantitative studies commonly evaluate the impact of vertical programs on a very limited set of outcomes, most often RI coverage [11]. Qualitative and quantitative evaluation methods are rarely robustly combined. Our research protocol thus advances an innovative model for mixed-methods research, aimed at evaluating the impact of vertical health programs, by: (a) combining quantitative and qualitative work into one coherent study design; (b) using purposively selected qualitative case studies to systematically evaluate the impact of key contextual variables; and (c) making extensive use of the method of participant observation to create comparative ethnographies of the impact of a single vertical program administered in varied contexts. This design allows the researcher to fill data gaps where the other method would be less well-suited, and provides a way to collect and analyze historical and context-rich data to inform future strategic evaluation.

The GPEI is a 20-year, $9-billion-dollar program that has harnessed the work of an estimated 20 million people in more than 100 countries [12,13]. Prior to our research, a number of studies had examined the impact of this program on health systems. Quantitative studies examined the impact of polio eradication on RI levels in India [14] and more broadly, in fifteen countries around the time polio eradication activities were initiated [15]. One major review of findings noted, "Quantitative studies on the effects of polio eradication on health systems development have been hampered by the lack of credible baseline data, the absence of control groups (areas that have not implemented polio eradication strategies), and the concurrent implementation of major health system reforms such as decentralization” [16]. These studies showed equivocal or mixed results; without qualitative contextual data, such results were difficult to conclusively explain or derive implications from.

A number of qualitative studies had also examined the impact of polio eradication, including immunization campaigns, surveillance, and other activities, on health systems. The earliest, based on interviews carried out in the Americas, showed that polio eradication activities had a positive impact in the Americas—where health systems were already strong [17]. Several of the researchers in that study cautioned that its findings might not apply to countries in Africa or Asia with weaker health systems [18]. Subsequent qualitative studies showed that polio eradication activities had positive effects in some areas and negative effects in others: for example, new health infrastructure was created in Lao PDR, but mass polio campaigns interfered with the timing of family planning activities in Nepal [19-21]. The authors of one study noted that the existing level of health service provision in a given country had important effects on the relationship between polio eradication and the health system [21]. The review cited above noted that overall, these studies provided rich context-specific information, but concluded that the results were "not readily generalizable" [16].

The study protocol described here is an attempt to garner a fine-grained, geographically broad and historically deep analysis of the effects of polio eradication activities on RI and PHC. Coordinated global polio eradication activities began in the 1990s in most of Africa and Asia, with most countries initially performing 2-3 polio vaccination campaigns per year. Our quantitative analyses used variation in the *intensity* of polio eradication activities (the number of campaigns per year) to get around the problem of a lack of control countries that have never carried out polio eradication activities. At the same time, we used qualitative approaches to determine *why* polio eradication activities had positively and negatively impacted RI and PHC in specific instances, identifying mediating factors, some of which are context-specific and others shared across different times and places. In our qualitative work, we paid special attention to the impact of concurrent health system reforms and other potential contextual confounders. We aimed to evaluate two hypotheses: (1) that the initial scale-up of polio eradication activities would lead to positive effects attributed to the creation of improved *quality* health systems; and (2) that increases in the intensity of the number of polio vaccination campaigns (to as many as 11 per year in parts of South Asia) would be tied to negative effects due to the *quantity* of work that the eradication of polio demanded, siphoning already-limited resources from the health system.

## Methods/Design

The study design described here grows out of a commitment to integrate qualitative and quantitative findings. Too often, qualitative data is seen as less substantive or definitive than quantitative results; qualitative work is undertaken only to supplement quantitative findings, not on its own merits. Our experience, described throughout this paper, indicates that both qualitative and quantitative approaches, undertaken systematically, have clear and substantial explanatory power. Vertical programs are of course planned and carried out by people—including doctors, surveillance officers, community health workers, and transnational monitors and experts, among others—and qualitative work has the power to illuminate the processes that drive human behavior in context. Also, as described below, our qualitative case studies were carefully selected to represent a wide range of contexts. This means that generalization from our qualitative data is to some degree possible: a trend that exists in all of our widely diverse case studies is likely to be a trend across much of South Asia and Africa. Further, since quantitative approaches are correlational and cannot prove causal relationships, we could turn to our qualitative case studies for information on causation.

Even when qualitative data do not lead to generalizable conclusions, such data are still extremely useful for understanding local processes of systemic change. And though quantitative data may lend themselves to generalizable conclusions (e.g., eradication activities have positive/negative/neutral impacts on health systems), policy-makers often need analyses that account for context-specific relationships and impacts. Thus mixed-methods research designs that truly integrate qualitative and quantitative findings are valuable tools.

The research protocol described here was approved by the Middlebury College IRB (proposal 11196).

### Case study selection

We carried out eight qualitative district-level case studies to examine the impact of the GPEI on RI and PHC. Because this relationship is complicated and highly context-dependent, these case studies aimed to provide an in-depth understanding of this relationship in the case study district, rather than in the country as a whole. The strengths of our qualitative approach depended on our researchers’ ability to gain as rich an understanding as possible of the complex processes they were asked to describe. Rather than attempt a whirlwind tour of a particular country covering much ground in little depth, our researchers had a chance to learn in some detail about the situation in a particular district, and can comment meaningfully about the ways in which eradication activities impact RI and PHC in that place. Districts were not meant to be representative of a country; our researchers’ familiarity with the countries in which they were working enabled them to put their particular study districts in context.

Our quantitative work, described below, analyzed the relationship of key variables between polio eradication campaigns, RI, and PHC in the same countries as our qualitative case studies, in addition to one global analysis.

Choosing the case studies was the first meeting point between our qualitative and quantitative methods. We selected our study districts equally from among the two major regions of ongoing polio transmission—South Asia and Sub-Saharan Africa. In a previous study, the relationship between polio eradication and other health services was affected by the general strength of health services in the area [21]. Therefore, our case studies were also selected to represent a range of levels of provision of RI and PHC, as determined by the most recent Demographic and Health Survey (DHS) studies available. Since DHS data allows regional-level but not district-level resolution, the above process led us to *regions* that fit our pre-defined criteria. Within those regions, final *districts* for case studies were selected by the case study researcher, often in conversation with relevant Ministry of Health officials in the country in question. We also selected case studies to represent a range of campaign intensities; our case studies experience as few as 2 to as many as 11 campaigns per year (see Table 1).

**Table 1:** Case study districts

**Case study district**	**Number of polio campaigns in 2011**	**Region**	**Attended births in region by TBA or higher**	**Antenatal care coverage in region**	**DTP3 coverage in region**
**Rautahat, Nepal**	4	Central Terai	58% (2006 DHS)	76% (2006 DHS)	91% (2006 DHS)
**Parba Champaran, Bihar, India**	9 to 10	Bihar	95% (2006 DHS)	34% (2006 DHS)	46% (2006 DHS)
**Nizamabad, Andhra Pradesh, India**	2	Andhra Pradesh	93% (2006 DHS)	96% (2006 DHS)	61% (2006 DHS)
**SITE Town, Karachi, Pakistan**	11	Sindh	93% (2007 DHS)	71% (2007 DHS)	48% (2007 DHS)
**South Omo, SNNP, Ethiopia**	2	SNNP	7% (2011 DHS)	42% (2011 DHS)	38% (2011 DHS)
**Kumbotso LGA, Kano, Nigeria**	8	Northwest	38% (2008 DHS)	32% (2008 DHS)	9% (2008 DHS)
**Rubavu, Rwanda**	2 to 3	Gisenyi	73% (2005 DHS)	93% (2005 DHS)	80% (2005 DHS)
**Camucuio, Namibe, Angola**	4	Sul	59% (2001 MICS)	83% (2001 MICS)	27% (2001 MICS)

### Quantitative research methods

We modeled the effects of polio eradication campaign intensity, and the maturity of polio eradication campaigns, on measures of RI and PHC within country using multivariate regression. We controlled for competing trends that may have also affected RI and PHC, such as other health care initiatives, increased funding, and political instability. The quantitative analyses illuminate broad patterns at large geographic scales that are not feasible to cover with detailed ethnographic studies, and give a sense of the degree to which case studies are representative of larger trends. Our general analytic strategy, tailored for each analysis, can be represented by the equation $H_{t2}\;=\;f\left(H_{t1},M,I,C\right)$ where: H_t2_ is the percentage coverage of a given measure of RI or PHC at the end of a given period of observation (e.g., a three-year period of time); H_t1_ is the percentage coverage of that measure at the beginning of the time period; M is the maturity of polio eradication efforts within a country as measured by the amount of time since the first house-to-house campaign; I is the intensity of polio eradication activities as measured by the number of campaigns within a given period of time; and C is a vector of contextual variables such as school enrollment rates, income levels, prevalence of atrocities, and regime type, as well as levels of external sources of RI funding and other health spending [22-27].

We performed analyses at a range of spatial scales: global, national, and district level. The global analysis used the nation as the unit of analysis, and included all countries for which campaign data were available. RI data were obtained from WHO/UNICEF [28,29] and the Institute for Health Metrics and Evaluation [26,30]. PHC was measured as attended birth coverage, obtained from the World Bank World Development Indicators [27]. The national time analysis examined our case study countries using subnational regions as units of analysis, with DHS data on RI and PHC indicators. District-level analyses were performed in select case study countries where data were available and used the district as the unit of analysis, with locally available data on RI and PHC indicators.

These different levels of analysis had complementary strengths and weaknesses. For the cross-national analysis, we could obtain annualized and reasonably up-to-date data for dependent, independent, contextual and likely covariate variables, but were unable capture the wide variation *within* countries in the number of campaigns per year, which likely diluted effects. On the other hand, in our sub-national analyses, we were able to match the spatial variability of polio eradication campaigns with spatial variability in changes of RI and PHC coverage measures. However, subnationally resolved data on RI, PHC, and polio eradication campaigns were frequently spotty, and many sources (for example, DHS surveys) were several years old. Despite these limitations, we found the quantitative analysis valuable for its ability to illuminate trends over time and across contexts.

### Qualitative research methods

Because standardization is critical in multi-sited qualitative research [31], the protocol was designed to facilitate collection of the same data in all eight district-level case studies to create a body of knowledge that was ethnographically rich but also systematically comparable [32]. The qualitative design of the study was based on three research strategies: (1) document review; (2) interviews; and (3) participant observation in district-level health activities including polio campaigns. To ensure that the same information was collected at all sites, a qualitative research guide provided the team with standardized research components and tasks, as well as a standardized list of deliverables. Once the first draft of the guide was developed, it was circulated amongst several of the country researchers and given some preliminary field-testing in Nepal and Ethiopia before being finalized.

First, researchers were provided with a specific list of documents relating to polio eradication, RI, and PHC to collect for each case study. These documents ranged from historical documents on national immunization policy to current street-by-street microplans for polio eradication campaigns in the study district. To allow for case sensitivity, researchers were encouraged to perform appropriate modifications and adaptations to the list. This proved effective—while researchers made minor necessary modifications, for the most part all researchers gathered comparable data (though a few had limitations described below).

Second, the interview section of the qualitative design included in-depth interviews with national-level health officials (n = 10 per case study), district-level health officials (n = 10 per case study), ground-level health staff at facilities and in communities (n = 30 per case study), and community members (n = 5 per case study). Again, the final list of interviewees was determined by the researchers according to the structure of the health system in each country, with the criteria to interview a range of officials, workers, and volunteers directly engaged in the administration of polio campaigns, RI, and PHC.

In-depth interviews were designed to explore diverse perspectives on mediators of the relationship between polio eradication activities, RI, and PHC. A detailed standardized interview guide was provided to all case study researchers, with over 30 questions, plus probes, designed to address specific aspects of this relationship. Informed consent was obtained from all participants.

Third, in each of the sites a case researcher conducted participant observation during polio eradication campaigns. As part of these participant observation activities, researchers attended all stages of the campaign (micro-planning, training, campaign implementation, catch-up, and monitoring and evaluation). Furthermore, researchers conducted clinic-centered participant observations in RI and PHC activities during and outside the campaign dates, and accompanied surveillance officers in their work. This gave researchers the opportunity to enhance data gathered during interviews and document review, uncovering how designs conceived at the global level get translated by national and local health administrations and carried out on the ground.

Each of the eight case studies, then, generated around 50 interview transcripts, participant observation fieldnotes, and a range of relevant technical and operational documents. These materials were coded with over 50 codes, selected to cover a wide variety of health system functions, using the qualitative analysis program NVivo. These included codes for surveillance; public awareness and satisfaction with health services; outreach to marginalized populations; cold chain; high-level government attention; and service interruption during campaigns, among others. When applicable, material in these categories was also coded as polio eradication, RI, or PHC to distinguish between the services offered by various programs. This allowed for comparisons and explorations of potential overlaps between, for example, outreach to marginalized populations during polio campaigns and outreach to the same populations for RI services. Such analyses revealed the positive and negative effects polio eradication had upon RI and PHC services.

It was central to our approach to collect nearly the same information at all eight research sites and to analyze it systematically and in parallel across the sites. This approach facilitated fairly direct comparisons of the ways that the Polio Eradication Initiative affected RI and PHC in various arenas at our different sites. We accomplished this in two ways. First, case study researchers prepared reports outlining their findings; these reports followed a standardized format covering potential impacts of polio eradication in nineteen health system domains, from surveillance to public satisfaction with health services. Second, after exhaustively coding all materials, we ran queries for our codes and prepared a spreadsheet summarizing the results of those queries for each study. This method allowed us to point out convergences and parallels, and to begin to tease apart the reasons for the differences in effects we saw across our field sites.

## Discussion

### Benefits of the design

This study design has four major benefits. First, the careful selection of a range of qualitative case studies allowed for systematic comparison across the studies. Second, in the qualitative work, the use of participant observation yielded important insights that interview and document review data alone might have missed. Third, the complementarity of qualitative and quantitative methods was very fruitful, as results from our quantitative analysis could be explained by results from qualitative work. Finally, this research provides useful information on how to implement vertical programs in the future in ways that will benefit health systems in the long run.

First, the use of multiple sites, carefully selected to represent a range of experiences with the GPEI, allowed us to distinguish between contextual and universal factors in the relationship between polio eradication, RI, and PHC. Our parallel case studies also allowed us to follow cross-cutting themes, such as cold chain, surveillance, and motivation of workers, across the different field sites, and reach conclusions regarding the role of these factors globally. For example, in a number of case studies—though not all of them—qualitative data supports the hypothesis that polio contributed to the development of cold chain infrastructure in the mid-90s. This was a subtle effect we could not pick up in analysis of the quantitative data available. The fact that it existed across such widely disparate case studies gives us strong grounds to suggest that the effect was widespread though not ubiquitous.

Other factors varied across the case studies, often in patterned ways. For example, in the very weak health systems in our study, polio eradication activities were often the only health activities occurring according to plan—in one case, the gound-level workers were on strike, and yet continued to work on polio vaccination campaigns. In such cases, polio eradication’s supervisory and incentive structure was one of the few motivations strong enough to get staff to work at all. In contrast, in many of the stronger health systems in our study, busy staff across all cadres juggled polio activities along with a variety of other responsibilities. A full understanding of the extent to which polio campaigns might or might not interrupt services must take such factors into account. The important patterned dynamics here—observable across widely disparate case studies in South Asia and Africa—were detected because of our systematic case study selection. This study design, then, allowed us to address general trends, as well as identify best practices in specific contexts that might be adapted for use in other places.

Second, in the qualitative portion of this research, participant observation yielded important insights that interview data alone might have missed. For example, when it came to topics like service interruption during campaigns, direct observation and experience with the dynamics described in the previous paragraph were often more illuminating than interviews, where respondents might be reluctant to speak frankly. By visiting local health posts both during campaign days and outside of campaigns, we were able to gather information on what constituted ‘normal’ health post functioning, and learn how that might or might not change during the campaign.

The third major benefit of the study design is that results from the quantitative analysis could be explained by results from qualitative work. In many cases, our quantitative analysis revealed no significant impact, either positive or negative, of the GPEI on RI or PHC. In these cases, the qualitative case studies often showed a complex mix of positive and negative effects, mediated through different factors, leading to an overall situation that could not be expected to have statistical significance in its impacts.

As an example of just one of many small effects, our qualitative data showed numerous examples of small-scale impact where supervision and monitoring and evaluation were performed at higher levels during the campaigns, and provided models for the health system that *could* be used for other services. (In many cases, however, they were not.) While too subtle to have a statistically observable impact on, say, DTP3 coverage, these areas of small impact visible through qualitative analysis are very important, and in some cases form the basis for recommendations for improvement.

Thus this research design has utility beyond simply assessing the impact of vertical programs on health systems in the past. Because the qualitative work revealed examples of best practices that could be more widely implemented, it also provides insight on ways that vertical programs like the GPEI could better strengthen health systems in the future. In addition, the rich description of on-the-ground strategies that comparative ethnography provides could be used to better understand how a given vertical program is being carried out in a variety of contexts, and could inform the development of new and improved program design. Further, in addition to providing currently actionable recommendations, the research described here could serve as groundwork for future research and strategic evaluation. For example, the quantitative and qualitative data that was collected in this research project could be used as baseline information to design more complex system dynamics models to test how various strategies might play out in the future [33].

### Challenges

As might be expected in carrying out parallel qualitative and quantitative analyses in eight case studies across the developing world, we experienced some challenges. However, we did identify ways to mitigate some of these challenges in the course of doing our research.

One of the major challenges confronted by every large-scale study in the developing world is the accessibility of data. In the quantitative arm of the study, we faced difficulties accessing reliable data for our analyses, particularly on where and when polio campaigns had occurred. In some countries such data did not exist, while in others government officials were reluctant to share them. Some officials may fear that analysis of quantitative data has the potential to reveal inadequacies in health services or otherwise reflect negatively on the health system; as long as this potential exists, officials may quite rationally decide that declining to share data is the safer course. In addition, discrepancies exist on immunization coverage estimates in particular and DHS, while robust, does not triangulate administrative data in proposing estimates. The short timelines allocated for the study, as well as the lack of political will and the need to locate the right partners in each of the study sites, made quantitative data collection extremely challenging—ultimately, despite our best efforts, impossible in a few cases (see Table 2).

**Table 2:** Availability of key data for quantitative analysis in study countries

	**Campaign data, sub-nationally resolved, from first campaigns to present**	**RI/PHC data at two timepoints**
**India**	Not provided by country. However, sufficient data was publicly available to make analysis possible.	DHS and other sources available
**Pakistan**	Available since 2005; provided by WHO/MoH	DHS and other sources available
**Nepal**	Available for all years; provided by WHO/MoH	DHS available
**Nigeria**	Available for all years in-country	DHS available
**Ethiopia**	Despite our best efforts, we were unable to access this data	DHS available
**Rwanda**	Despite our best efforts, we were unable to access this data	DHS available
**Angola**	Despite our best efforts, we were unable to access this data	Not to our knowledge available; no DHS and MICS with two comparable time points in last 10 years
**Global analysis**	Available, resolved at country level, from WHO Geneva	Available (e.g., UNICEF and IHME)

The quantitative analyses were further complicated by the nature of polio vaccination campaigns themselves. The targeting of campaigns has changed over time. While early efforts were almost exclusively national in scope, over time, targeting evolved to focus on first level administrative units (states/provinces) and then to second level administrative units (districts). As targeting became more sophisticated, regions with relatively low levels of RI and PHC coverage had the most frequent polio eradication campaigns. This creates a selection bias that makes it very difficult to establish causality between the frequency of polio eradication campaigns and changes in RI and PHC coverage.

To avoid confounders and address selection bias, a potential way forward is for a study to collect new quantitative data on key variable(s) of interest—depending on the vertical program being studied, possibilities include functioning and reach of the cold chain, number of people serviced per day for a specific service (e.g., routine immunization) by type of worker, or availability of key supplies like syringes. Such a study design might also allow the quantitative analysis to pick up small impacts on cold chain or worker time availability that might not be strong enough to affect a downstream indicator like overall DTP3 coverage. Creating such datasets would, of course, require significant time and resources. Ideally, these efforts would be designed into and funded as part of the evaluation process for vertical campaigns.

The collection of qualitative data was challenged by accessibility as well. In-country approval procedures including Institutional Review Boards (IRBs) are continually evolving and becoming more complex, and the steps required are not always clear. While we congratulate the formation of institutional and national IRBs, this change in procedure requires specific attention. In addition, it is increasingly difficult for research projects to compete with donors and programs that bring direct funding and benefits to government officials. Methodological advancement on this front is not simple, and ultimately requires improving relations between country officials and researchers. Before providing approval for a research project that required time on the part of government officials and health workers, we found that government officials reasonably wanted to know how they and their programs would benefit from the research. Therefore, researchers need to be very clear about how their research may be used to the country’s benefit, and transparent about how and with whom the results of the study will be shared.

We recommend assigning a local partner to the position of a full-time in-country point-person. Such a point-person would not be engaged in actual data collection, but would be responsible for nurturing political will, creating partnerships, and leading the study’s interactions with local IRBs. While such tasks were in the past do-able in a short time span, or even from afar, the situation on the ground in most countries today necessitates a full-time staff member to liase with local institutions and policies.

Further, any multi-sited study involving a variety of countries has potential to encounter problems of security and political instability; for example, we faced political unrest in our case studies in Kano, Nigeria and Karachi, Pakistan. Our study’s qualitative data collection team struggled with a constant need to adapt to changing circumstances in order to ensure the personal safety of our staff. We accepted that the standardized research protocol would sometimes have to be modified to meet the exigencies of fieldwork. While ideally we would have collected identical data in all eight case studies, security issues and other extenuating circumstances limited our ability to follow exactly the same guidelines in all of the sites. For example, when our researcher in Karachi, Pakistan received a text message warning her not to go to the study site as she might be targeted, she scaled back her participant observation activities from those outlined in the guide. Such deviations are inevitable in a project committed to gathering data in a range of contexts, not just settings where research is convenient.

Beyond accessibility, in a large research project there is likely to be variability (including among researchers working at the same site) in the experience and skill level of qualitative field researchers. In the course of this research we learned that our more experienced fieldworkers found it easier to adapt our research protocol to local situations. These experienced staff appreciated having the leeway to make their own adaptations to the research tasks, and those of us running the study valued their thoughtful, context-appropriate modifications.

Those research staff with less experience found it more challenging to make necessary modifications to the research guide to tailor it to local conditions. To support their work, we recommend (1) providing samples of appropriate adaptations in the research guide; (2) pairing less experienced researchers with more experienced researchers when possible; and (3) holding a series of orientation sessions for all research staff prior to conducting the research, to discuss questions of appropriate modifications.

We did not hold such orientation sessions, but would do the following in the future: The first orientation session would include the lead researcher for each case study and would be dedicated to refining the research guide and discussing questions regarding the methods to be used in the study. This orientation should be held after a pilot, testing interview questions in the field, in at least one study site. A second pre-fieldwork workshop would be held in-country, and would include the full team of fieldworkers for that country. The in-country workshop would be used to test interview questions in a short country-specific pilot and make the appropriate country-specific adjustments to the study’s methods and tasks prior to fieldwork. Periodic debriefs among field staff over the course of fieldwork, concentrated in the early stages, would ensure that staff had the opportunity to discuss challenges and provide suggestions for appropriate solutions in modifying the guide. We held a workshop of fieldworkers post-fieldwork and found this to be an extremely useful exercise in discussing cross-cutting themes and results.

Finally, in developing a research protocol, we would encourage the oversampling of historically knowledgeable key informants. In many of our case studies, there were too few interviewees with deep historical memory. We found that frequent transfers limited the number of respondents with institutional memory, including locating documents and quantitative data from early years. Those who did have such knowledge were generally the most useful interviewees. As detailed quantitative data from the early years of the GPEI was often unavailable, rich qualitative data illuminating past trends is especially important. Interview protocols should also pay special attention to the need to learn about events in the past.

Flexibility in research across sites includes the ability to make adjustments to the timeline and budget to adapt to changing circumstances. While most public health research is held to short timelines because of the need to make data-driven programmatic decisions, more time allocated to research projects will mean better quality research. Flexibility in budget planning is important as well. The challenges of data accessibility, the need to realign to meet new IRB procedures, and the need to address changes in political circumstances, are easiest to productively address in the context of a flexible timeline and a flexible budget.

## Conclusion

The study protocol described here has the potential to be adapted and more widely used in studies of the effects of vertical programs on health systems. Overall, our study design yielded rich data that can be compared across a diversity of sites and could provide important foundational work for future evaluations or models. Drawing from our experience in this study, we encourage researchers to establish mixed-methods research designs in which qualitative data is used not just to embellish quantitative results, but rather uses both types of data as integral parts of the analysis. Using qualitative and quantitative methods together, including participant observation, and making use of comparative ethnography are useful tools for crafting health systems research that is attentive to local context even as it has the ability to describe global trends.

## Abbreviations

DHS: Demographic and Health Survey; DTP3: Third dose of the diphtheria-tetanus-pertussis vaccine; GAVI: Global Alliance for Vaccines and Immunizations; GPEI: Global Polio Eradication Initiative; IHME: Institute for Health Metrics and Evaluation; IRB: Institutional Review Board; MICS: Multiple Indicator Cluster Survey; LGA: Local Government Area; MoH: Ministry of Health; PHC: Primary Health Care; RI: Routine Immunization; SITE: Sindh Industrial and Training Estate; SNNP: Southern Nations, Nationalities, and People's Region; TBA: Traditional Birth Attendant; UNICEF: United Nations Children’s Fund; WHO: World Health Organization.

## Competing interests

All authors declare they have no competing interests.

## Authors' contributions

SC, TP, KM, JJ, MAL, RML, and JK developed the original concept for the research design. AR and SC developed the detailed qualitative research protocol. TP, MAL, and RML developed the detailed quantitative research design. KM and JJ field tested and provided feedback on the qualitative research protocol. JG, LV, and PN provided extensive feedback that substantively changed the study design. KC, PT, and SC incorporated all feedback and finalized the qualitative research protocol. KC, PT, and SC developed the coding system for qualitative data. SC and AR drafted the article, with sections written by VN and TP. All listed authors provided extensive feedback on the article, and read and approved the final manuscript.

## Pre-publication history

The pre-publication history for this paper can be accessed here:

http://www.biomedcentral.com/1471-2458/12/728/prepub

## References

[B1] Cueto M The origins of primary health care and selective primary health care Am J Public Health 2004 94 1864 1874 10.2105/AJPH.94.11.1864 15514221 PMC1448553

[B2] Garrett L The challenge of global health Foreign Affairs 2007 86 14

[B3] Bosch-Capblanch X Lavis JN Lewin S Atun R Røttingen J-A Dröschel D Beck L Abalos E El-Jardali F Gilson L Oliver S Wyss K Tugwell P Kulier R Pang T Haines A Guidance for evidence-informed policies about health systems: rationale for and challenges of guidance development PLoS Med 2012 9 e1001185 10.1371/journal.pmed.1001185 22412356 PMC3295823

[B4] Lavis JN Røttingen J-A Bosch-Capblanch X Atun R El-Jardali F Gilson L Lewin S Oliver S Ongolo-Zogo P Haines A Guidance for evidence-informed policies about health systems: linking guidance development to policy development PLoS Med 2012 9 e1001186 10.1371/journal.pmed.1001186 22427746 PMC3302830

[B5] Lewin S Bosch-Capblanch X Oliver S Akl EA Vist GE Lavis JN Ghersi D Røttingen J-A Steinmann P Gulmezoglu M Tugwell P El-Jardali F Haines A Guidance for evidence-informed policies about health systems: assessing how much confidence to place in the research evidence PLoS Med 2012 9 e1001187 10.1371/journal.pmed.1001187 22448147 PMC3308931

[B6] Glassman A Chalkidou K Priority-Setting in Health: Building Institutions for Smarter Public Spending 2012 Washington, DC: Center for Global Development

[B7] Cavalli A Bamba S Traore M Boelart M Coulibaly Y Polman K Pirard M Dormael M Interactions between global health initiatives and country health systems: the case of a neglected tropical diseases control program in Mali PLoS Negl Trop Dis 2010 4 e798 10.1371/journal.pntd.0000798 20808908 PMC2923152

[B8] Coulibaly Y Cavalli A van Dormael M Polman K Kegels G Programme activities: a major burden for district health systems? Trop Med Int Health 2008 13 1430 1432 10.1111/j.1365-3156.2008.02174.x 18983273

[B9] The Maximizing Positive Synergies Academic Consortium Interactions between Global Health Initiatives and Health Systems: Evidence from Countries 2009 Geneva: World Health Organization

[B10] Hanvoravongchai P Mounier-Jack S Oliveira Cruz V Balabanova D Biellik R Kitaw Y Koehlmoos T Loureiro S Molla M Nguyen H Ongolo-Zogo P Sadykova U Sarma H Teixeira M Uddin J Dabbagh A Griffiths UK Impact of measles elimination activities on immunization services and health systems: findings from six countries J Infect Dis 2011 204 S82 S89 10.1093/infdis/jir091 21666218

[B11] Verguet S Jassat W Hedberg C Tollman S Jamison DT Hofman KJ Measles control in Sub-Saharan Africa: South Africa as a case study Vaccine 2012 30 1594 1600 10.1016/j.vaccine.2011.12.123 22230581

[B12] World Health Organization Global Polio Eradication Initiative Strategic Plan 2004–2008 2003 Geneva: WHO

[B13] World Health Organization UNICEF: Global Polio Eradication Initiative Financial Resource Requirements 2012–2013 As of 1 May 2012 2012 Geneva: WHO

[B14] Bonu S Rani M Baker T The impact of the national polio immunization campaign on levels and equity in immunization coverage: evidence from rural North India Soc Sci Med 2003 57 1807 1819 10.1016/S0277-9536(03)00056-X 14499507

[B15] Bonu S Rani M Razum O Global public health mandates in a diverse world: the polio eradication initiative and the expanded programme on immunization in Sub-Saharan Africa and South Asia Health Policy 2004 70 327 345 10.1016/j.healthpol.2004.04.005 15488998

[B16] Loevinsohn B Aylward B Steinglass R Ogden E Goodman T Melgaard B Impact of targeted programs on health systems: a case study of the polio eradication initiative Am J Public Health 2002 92 19 23 10.2105/AJPH.92.1.19 11772750 PMC1447377

[B17] Pan American Health Organization The Impact of the Expanded Program on Immuniztion and the Polio Eradication Initiative on Health Systems in the Americas: Final Report of the “Taylor Commission” 1995 Washington, DC: PAHO

[B18] Taylor CE Cutts F Taylor ME Ethical dilemmas in current planning for polio eradication Am J Public Health 1997 87 922 925 10.2105/AJPH.87.6.922 9224170 PMC1380923

[B19] Dietz V Cutts F The use of mass campaigns in the expanded program on immunization: a review of reported advantages and disadvantages Int J Health Serv 1997 27 767 790 10.2190/QPCQ-FBF8-6ABX-2TB5 9399118

[B20] World Health Organization Meeting on the Impact of Targeted Programmes on Health Systems: A Case Study of the Polio Eradication Initiative 2000 Geneva: WHO

[B21] Mogedal S Stenson B Disease Eradication: Friend or Foe to the Health System? 2000 Geneva: WHO 29

[B22] GAVI Alliance Disbursements by countryhttp://www.gavialliance.org/results/disbursements/

[B23] World Health Organization Global Health Observatory Data Repositoryhttp://apps.who.int/ghodata/#

[B24] Marshall MG Jaggers K Gurr TR Polity IV Project: Political Regime Characteristics and Transitions, 1800–2010. Dataset User’s Manual 2011 Vienna, Virginia: Center for Systemic Peace www.systemicpeace.org/polity/polity4.htm

[B25] Political Instability Task Force Worldwide Atrocities Datasethttp://eventdata.psu.edu/data.dir/atrocities.html

[B26] Institute for Health Metrics and Evaluation Development assistance for health country and regional recipient level database 1990–2009 http://www.healthmetricsandevaluation.org/ghdx/record/development-assistance-health-country-and-regional-recipient-level-database-1990-2009

[B27] World Bank World Development Indicators http://api.worldbank.org/datafiles/SH.STA.BRTC.ZS_Indicator_MetaData_en_EXCEL.xls

[B28] Burton A Monasch R Lautenbach B Gacic-Dobo M Neill M Karimov R Wolfson L Jones G Birmingham M WHO and UNICEF estimates of national infant immunization coverage: methods and processes Bull World Health Organ 2009 87 535 541 10.2471/BLT.08.053819 19649368 PMC2704038

[B29] World Health Organization UNICEF WHO/UNICEF Coverage Estimateshttp://www.who.int/entity/immunization_monitoring/data/coverage_estimates_series.xls

[B30] Lim SS Stein DB Charrow A Murray CJ Tracking progress towards universal childhood immunisation and the impact of global initiatives: a systematic analysis of three-dose diphtheria, tetanus, and pertussis immunisation coverage Lancet 2008 372 2031 2046 10.1016/S0140-6736(08)61869-3 19070738

[B31] Griffiths UK Hanvoravongchai P Oliveira Cruz V Mounier-Jack S Balabanova D A Toolkit for Assessing the Impacts of Measles Eradication Activities on Immunization Services and Health Systems at Country Level 2010 London: London School of Hygeine and Tropical Medicine

[B32] Hirsch JS Smith DJ Phinney HM Parikh S Nathanson C The Secret: Love, Marriage, and HIV 2010 Nashville, TN: Vanderbilt University Press

[B33] Homer JB Hirsch GB System dynamics modeling for public health: background and opportunities Am J Public Health 2006 96 452 458 10.2105/AJPH.2005.062059 16449591 PMC1470525

